# Electro-Puncture

**Published:** 1843-06

**Authors:** 


*Electro-Puncture.*—Schuster has lately addressed a paper to the French Academy of Sciences, to prove that electricity is little serviceable in medicine unless it be applied through the means of acupuncture needles. Administered in this way, he asserts it may be employed with success against dropsies of all kinds, steatomatous and other tumours, engorgements and indurations (and perhaps cancer,) goitre, varicose dilatations, chronic rheumatism, paralysis, amaurosis, and perhaps aneurism. Electro-puncture acts, he says, by directly stimulating the sensibility, contractility, and absorbent function; in the formation of internal eschars, and the detraction, piece by piece, of diseased growths; in decomposing the fluid contents of tumours; in procuring external openings for matter, provoking adhesive inflammations, &c.; though some of these effects seem capable of production by the needles alone without the electricity.—*Lond. Lancet.*

